# Impact of the improvement of living conditions on tuberculosis mortality in Brazil: an ecological study

**DOI:** 10.1590/1516-3180.2023.0279.R1.13052024

**Published:** 2024-08-26

**Authors:** Marcio Natividade, Marcos Pereira, Christine Stauber, Samilly Miranda, Maria Glória Teixeira, Ramon Andrade de Souza, Marilia Santos dos Anjos, Rafael Barros, Daniela Gonçalves Morato, Erika Aragão, Susan Martins Pereira, Maria da Conceição Nascimento Costa

**Affiliations:** I Adjunct Professor, Department of Public Health, Institute of Collective Health, Universidade Federal da Bahia (UFBA), Salvador (BA), Brazil.; II Adjunct Professor, Department of Public Health, Institute of Collective Health, Universidade Federal da Bahia (UFBA), Salvador (BA), Brazil.; III Professor, Department of Population Health Sciences, School of Public Health, Georgia State University, Atlanta, Georgia, United States.; IV Adjunct Professor, Department of Public Health, Institute of Collective Health, Universidade Federal da Bahia (UFBA), Salvador (BA), Brazil.; V Department of Public Health, Institute of Collective Health, Universidade Federal da Bahia (UFBA), Salvador (BA), Brazil.; VI Nurse, Postgraduate Program in Collective Health, Institute of Collective Health, Universidade Federal da Bahia (UFBA), Salvador (BA), Brazil.; VII Nurse, Postgraduate Program in Collective Health, Institute of Collective Health, Universidade Federal da Bahia (UFBA), Salvador (BA), Brazil.; VIII Adjunct Professor in Nursing School, Universidade Federal da Bahia (UFBA), Salvador (BA), Brazil.; IX Bachelor of Science in Biological Sciences, Institute of Collective Health, Universidade Federal da Bahia (UFBA), Salvador (BA), Brazil.; X Associate Professor, Department of Public Health, Institute of Collective Health, Universidade Federal da Bahia (UFBA), Salvador (BA), Brazil.; XI Associate Professor, Department of Public Health, Institute of Collective Health, Universidade Federal da Bahia (UFBA), Salvador (BA), Brazil.; XII Associate Professor, Department of Public Health, Institute of Collective Health, Universidade Federal da Bahia (UFBA), Salvador (BA), Brazil.

**Keywords:** Tuberculosis, Social Conditions, Spatio-Temporal Analysis, Living Conditions, Tuberculosis Mortality Rate, Mortality

## Abstract

**BACKGROUND:**

The risk of death due to tuberculosis (TB) in Brazil is high and strongly related to living conditions (LC). However, epidemiological studies investigating changes in LC and their impact on TB are lacking.

**OBJECTIVES:**

To evaluate the impact of LC on TB mortality in Brazil.

**DESIGN AND SETTING:**

This ecological study, using panel data on spatial and temporal aggregates, was conducted in 1,614 municipalities between 2002 and 2015.

**METHODS:**

Data were collected from the Mortality Information System and the Brazilian Institute of Geography and Statistics. The proxy variable used for LC was the Urban Health Index (UHI). Negative binomial regression models were used to estimate the effect of the UHI on TB mortality rate. Attributable risk (AR) was used as an impact measure.

**RESULTS:**

From 2002 to 2015, TB mortality rate decreased by 23.5%, and LC improved. The continuous model analysis resulted in an RR = 0.89 (95%CI = 0.82–0.96), so the AR was −12.3%. The categorized model showed an effect of 0.92 (95%CI = 0.83–0.95) in municipalities with intermediate LC and of 0.83 (95%CI = 0.82–0.91) in those with low LC, representing an AR for TB mortality of −8.7% and −20.5%, respectively.

**CONCLUSIONS:**

Improved LC impacted TB mortality, even when adjusted for other determinants. This impact was greater in the strata of low-LC municipalities.

## INTRODUCTION

Tuberculosis (TB) is associated with poverty and poor living conditions (LC), especially in low- and middle-income countries.^1^ This neglected disease requires strategies that consider humanitarian, economic, and public health aspects for its control.^2,3^

Globally, the incidence of TB has fallen by approximately 2% annually between 2015 and 2020, with a cumulative reduction of 11%.^4^ However, the COVID-19 pandemic has contributed to an increase in the number of cases and increasing TB mortality in some countries. In 2022, an estimated d 10.6 million people developed active TB compared to 10,1 million in 2020.^4^ The incidence rate of TB increased by 3.9% between 2020 and 2022, suggesting a reversal from the trend of nearly 2% decrease per year during the past two decades. In addition, 1.6 million deaths from the disease, compared with 1.5 million in 2020,^4^ and TB is the third leading cause of death due to infectious diseases and the first among patients diagnosed with human immunodeficiency virus (HIV).^5^

Brazil has the highest number of reported TB cases in the Americas.^6^ In 2019, tuberculosis incidence and mortality in Brazil were estimated as 46 and 3.3 per 100,000 population, respectively.^7^ From 2011 to 2015, this coefficient had an annual percentage change of −1.9%, followed by an increase of 2.4% until 2019.^8^ In 2022, Brazil recorded 81,000 new TB cases, corresponding to an incidence rate of 32.0 cases per 100,000 population.^9^

Most TB deaths primarily occur in low-income countries, and their incidence is associated with precarious living conditions, especially precarious living and work conditions, including overcrowding and inadequate ventilation. These results highlight TB as a serious public health problem, characterizing it as one of the infectious diseases with the highest mortality rates in the world.^1,10^

According to the United Nations Sustainable Development Goals (2016-2030), the 90-90-90 targets for TB involve monitoring 90% of vulnerable populations, diagnosing and starting treatment in 90% of cases, curing at least 90% of these, reducing the number of families affected by TB to zero, and facing catastrophic costs due to the disease.^11^

Social protection interventions aimed at reducing social inequalities and improving the LC of vulnerable populations can contribute to controlling TB and containing the epidemic.^12,13^ This is because social protection interventions synergistically affect treatment results owing to improvements in nutritional conditions, psychosocial health, and access to health services.^13,14^

Epidemiological studies have reported that the implementation of public social policies in the last 15 years, focusing on the poorest population in Brazil, has improved the LC of the population, which has produced favorable effects on some health problems.^15,16^ Despite this evidence, no studies have been conducted on the impact of LC on TB mortality, considering Brazil as a whole.

## OBJECTIVE

This study aimed to verify the impact of LC on TB mortality in Brazil between 2002 and 2015.

## METHODS

### Data, population, and sources

A longitudinal ecological study was conducted with panel data on multiple spatial and temporal aggregates, using Brazilian municipalities and calendar year as units of analysis between 2002 and 2015.

Of the 5,570 municipalities in Brazil, 1,614 (28.9%) were selected, whose vital records (death and birth information) presented satisfactory quality, according to the criteria adopted by Andrade and Szwarcwald^17^ and Rasella et al.,^15^ as follows: average relative deviation of the general mortality coefficient ≤ 20.0 for municipalities with < 50 thousand inhabitants; ≤ 6.1 for municipalities with ≥ 50 thousand inhabitants; proportion of live births reported and estimated ≥ 0.9 for municipalities with < 50 thousand inhabitants; ≥ 0.7 for municipalities with ≥ 50 thousand inhabitants; mean deviation relative to birth rate ≤ 17.1 for municipalities with < 50 thousand inhabitants; ≤ 8.1 for municipalities with ≥ 50 thousand inhabitants; and proportion of deaths without definition of the basic cause ≤ 20.7 for municipalities with < 50 thousand inhabitants; ≤ 16.2 for municipalities with ≥ 50 thousand inhabitants.^15,17^

### Variables of the study and measurement

The number of deaths from all forms of TB (codes A15 – A19 in the International Classification of Diseases, 10th revision) was obtained from the Mortality Information System, and the population of the municipalities was extracted from the databases (including interpolation and extrapolation estimates) made available by the Brazilian Institute of Geography and Statistics.^18^ The annual TB mortality rate was calculated from the ratio between the total number of TB deaths and the population of the municipality multiplied by 100,000 inhabitants.

Variables known as potential determinants of TB mortality were selected based on their availability and relevance. They were used in the statistical analysis, continuously and categorically, as follows: TB-HIV coinfection rate ("0" < 5.0% and "1" ≥ 5.0%); TB treatment dropout rate ("0" < 5.0% and "1" ≥ 5.0%); coverage of the Family Health Strategy (FHS) ("0" ≥ 30.0% and "1" < 30.0%); TB cure rate ("0" < 10.0% and "1" ≥ 10.0%); rate of hospitalization for TB/100 thousand inhabitants ("0" < 10.0% and "1" ≥ 10.0%); proportion of older men – 65 years and over ("0" < 4.0% and "1" ≥ 4.0%). These data were obtained from the Department of Informatics of the Brazilian Unified Health System of the Ministry of Health.^19^

The variable used as a proxy for LC was the "Urban Health Index" (UHI), also used in continuous and categorical modeling, and it was stratified into tertiles: first tertile (< 0.278) high LC; second tertile (≥ 0.278 and < 0.330) intermediate LC; and third tertile (≥ 0.330) low LC. This composite indicator allows for a flexible approach to the selection, compilation, and presentation of data in the health field to graphically and visually show statistical health inequalities.^20–22^

To construct this index, the following indicators were selected for each municipality: population of low-income people (proportion of residents with a monthly household income per capita of up to 1/2 minimum wage); income per capita (monthly household income per person); black population (proportion of black people); illiteracy rate (proportion of people aged 15 and over who are illiterate); schooling rate (proportion of people with 15 and more years of study); piped water (proportion of households connected to a regular water supply network); garbage collection (proportion of households with regular garbage collection); household density (average number of people per household); unemployment rate (proportion of economically active people who are unemployed, per 100 inhabitants); GDP per capita (gross domestic product per 100 inhabitants living in the municipality); and health establishments (proportion of basic healthcare establishments per 100 inhabitants).

After their selection, these indicators were classified in ascending order for those where higher values indicated worse LC and in descending order for those where the higher the value, the worse the situation. Mathematical standardization of the indicators was then performed,^20^ followed by their combination and calculation of the geometric mean for each municipality, using the tool to calculate the UHI.^20^ The result of which is an adapted score that varies from 0 to 1 for each area, in which the closer to 1 or higher this score is, the worse the LC of the population of that municipality.^22^

### Variables of the study and measurement

The evolution of the mean annual TB mortality rates, UHI, and selected covariates is described. The effects of the UHI (crude and adjusted) on the mean mortality rate were estimated using negative (continuous and categorical) binomial regression models for panel data with fixed effects specifications for the covariates mentioned in the selected municipalities through risk ratio (RR) estimates.

The choice between fixed and random effects was based on the Hausman test, which evaluates the differences in the estimates of the two effects.^23,24^ For the evaluation of public policies, the fixed-effects model is the most appropriate, as it allows the control of unobserved variables that are constant over time (geographic and sociocultural characteristics of the municipality), which can be correlated with the independent variables, controlling the bias prior to the implementation of the programs. These analyses were performed using the Stata software version 15 (StataCorp LLC., College Station, Texas, United States).

The following equation expresses the panel data regression model, where municipalities are represented by subscript i and years by subscript t.


$${\text{TB}}_{\text{it}}=\text{β}1{\text{UHI}}_{\text{it}}+{\text{βX}}_{\text{it}}+{\text{α}}_{\text{i}}+{\text{u}}_{\text{it}}$$

TB*it*: Logarithm of the mortality coefficient for tuberculosis in municipality i in year t.

β1UHI*it*: Level of the Urban Health Index in municipality i in year t.

βX*it*: Value of each covariable included in municipality i in year t;

α*i*: Fixed effect for municipality i that captures all the unobserved characteristics that vary in time;

u*it*: Regression error.

To verify the contribution of the improvement in LC to reducing the relative risk of TB mortality, we used attributable risk (AR).^25^

### Ethical approval

This study was approved by the Ethics Committee for Research Involving Human Beings of the Institute of Collective Health of the Universidade Federal da Bahia (No. 1,527,799) on May 3, 2016. All study procedures were performed in accordance with the Code of Ethics of the World Medical Association (Declaration of Helsinki) for experiments involving humans. Informed consent for experimentation with human participants and their privacy rights were not required owing to the use of secondary data.

## RESULTS

### Mortality due to tuberculosis and other causes in Brazil

Between 2002 and 2015, 65,148 (2.4/100,000 inhabitants) TB deaths were documented in Brazil. In the 1,614 municipalities analyzed, this number was 26,336 (2.8/100,000 inhabitants), corresponding to 40.4% of the total number of TB deaths. There was a decline in the mortality rate from this disease (23.5 %), varying from 3.4/100,000 in 2002 to 2.6/100,000 inhabitants in 2015 (Table 1).

**Table 1:** Tuberculosis,^1^ annual number of deaths (n) and mortality rate (MR/1,000 inhabitants) for Brazil and a set of municipalities selected for the study, from 2002 to 2015

Year	Brazil		Selected municipalities^2^		
	n	MR	n	%^3^	MR
2002	5,048	2.8	2,149	42.6	3.4
2003	4,843	2.7	2,007	41.4	3.2
2004	4,838	2.6	2,002	41.4	3.1
2005	4,602	2.5	1,868	40.6	2.9
2006	4,721	2.5	1,882	30.9	2.9
2007	4,612	2.4	1,781	38.6	2.7
2008	4,756	2.5	1,908	40.1	2.8
2009	4,690	2.4	1,877	40.0	2.8
2010	4,568	2.3	1,801	39.4	2.6
2011	4,460	2.3	1,780	39.9	2.6
2012	4,316	2.2	1,758	40.7	2.5
2013	4,617	2.3	1,856	40.2	2.6
2014	4,467	2.2	1,841	41.2	2.6
2015	4,610	2.3	1,826	39.6	2.6
**Variation (%)**	**-8.7**	**-17.9**	**-15.0**	**--**	**-23.5**
**Total**	**65,148**	**2.4**	**26,336**	**40.4**	**2.8**

Source: Mortality Information System. SINAN / Datasus / Ministry of Health.

1 All forms;

2 They refer to 1,614 municipalities that had better information quality;

3 Percentage of the total number of municipalities in Brazil; MR = mortality rate.

The mean UHI value was 0.309 (0.126–0.541). In 2002, this index was 0.323; in 2015, it was 0.296, an average reduction of 8.4% in the selected municipalities. On average, 4.1% of the TB-notified individuals in the municipalities included in the study were HIV-infected, an increase of 17.8% (3.9% in 2002 to 4.6% in 2015). The treatment dropout rate decreased by 51.5% during this period, with an overall average rate of 5.2%. Among the 1,614 municipalities, a 52.6% increase was observed in the average coverage of FHS, with an average coverage ratio of 64.7%. The mean cure rate for this disease is 61.7%. Hospitalizations for TB decreased by 46.7% over the study period, with an average of 4.9/100,000 inhabitants. The proportion of older adults living in these areas was 3.9%, which increased by an average of 39.9% during the study period (Table 2).

**Table 2:** Average annual values of the Urban Health Index (UHI), proportion of demographic indicators and health care variation (%), and average in the period. Brazil^1^ 2002–2015

Years/Indicators	UHI	% TB-HIV coinfection ratio	% TB treatment dropout	% coverage FHS	% cure TB	Rate hospitalization TB^2^	% older men
**2002**	0.323	3.9	6.2	46.6	61.9	6.4	3.3
**2003**	0.321	3.0	5.4	51.9	61.3	6.7	3.3
**2004**	0.318	3.2	6.8	56.2	65.1	6.0	3.4
**2005**	0.316	3.3	4.8	60.6	60.5	5.4	3.5
**2006**	0.314	3.5	4.8	64.2	58.6	5.3	3.5
**2007**	0.312	4.1	4.9	67.0	61.8	4.5	3.6
**2008**	0.310	4.1	5.4	68.0	61.5	8.9	3.7
**2009**	0.308	4.8	5.2	68.6	62.3	3.7	3.8
**2010**	0.306	4.7	5.1	69.9	63.8	3.8	4.0
**2011**	0.304	5.1	5.5	69.3	60.8	3.8	4.1
**2012**	0.302	5.2	5.4	70.4	62.2	3.8	4.2
**2013**	0.300	4.1	5.8	71.1	60.3	3.7	4.4
**2014**	0.298	4.4	4.5	71.2	63.4	3.4	4.6
**2015**	0.296	4.6	3.0	71.1	60.1	3.4	4.6
**Variation (%)**	**-8.4**	**17.8**	**-51.5**	**52.6**	**-3.0**	**-46.7**	**39.9%**
**Average**	**0.309**	**4.1**	**5.2**	**64.7**	**61.7**	**4.9**	**3.9**

Source: Mortality Information System. SINAN / Datasus / Ministry of Health.

1 They refer to 1,614 municipalities that had better information quality.

UHI = Urban Health Index; TB = tuberculosis; HIV = human immunodeficiency virus; FHS = Family Health Strategy.

### Living conditions and tuberculosis mortality

In the analysis with the continuous and adjusted modeling for the selected covariates, a statistically significant overall protective effect of UHI on TB mortality was observed, with an RR = 0.89 (95%CI = 0.82–0.96), and the AR was −12.3% (Table 3). For the model with categorized variables, Table 4 shows that UHI was also associated with TB mortality rate. For municipalities with intermediate LC, the effect was 0.92 (95%CI = 0.83–0.95), and in those with low LC, it was 0.83 (95%CI = 0.82–0.91); that is, the AR for TB mortality was −8.7% and −20.5%, respectively.

**Table 3:** Estimated relative risk (RR) for the association between tuberculosis mortality rate^1^ and Negative Binomial Regression Urban Health Index.^2^ Brazil^3^ 2002–2015

Variables	Model			
	Crude		Adjusted	
	RR	95%CI	RR	95%CI
Urban Health Index	0.72	**0.66–0.77**	**0.89**	**0.82–0.96**
TB-HIV coinfection ratio			1.01	1.01–1.05
Proportion of TB treatment dropout			1.00	1.00–1.04
Coverage of the Family Health Strategy			0.97	0.97–0.99
TB cure rate			1.00	1.00–1.01
Rate of hospitalization TB			1.20	1.00–1.33
Proportion of older men – 65 years and over			1.81	1.80–1.83

Source: Mortality Information System. SINAN / Datasus / Ministry of Health.

1 All forms;

2 Continuous model;

3 They refer to 1,614 municipalities with better information quality.

RR = relative risk; CI = confidence interval.

**Table 4:** Estimated relative risk (RR) for the association between tuberculosis mortality rate^1^ and Urban Health Index obtained through Negative Binomial Regression.^2^ Brazil^3^ 2002–2015

Variables		Model			
		Crude		Adjusted	
		RR	95%CI	RR	95%CI
**Urban Health Index**					
	1° Tercil (< 0.278) High LC	**1**	**-**	**1**	**-**
	2° Tercil (≥ 0.278 e < 0.330) Intermediate LC	**0.95**	**0.87–0.99**	**0.92**	**0.83–0.95**
	3° Tercil (≥ 0.330) Low LC	**0.89**	**0.82–0.98**	**0.83**	**0.82–0.91**
**TB-HIV coinfection ratio**					
	< 5.0%	-	-	1	-
	≥ 5.0%	-	-	1.32	1.24–1.42
**Proportion of TB treatment dropout**					
	< 5.0%	-	-	1	-
	≥ 5.0%	-	-	1.33	1.25–1.42
**Coverage of the Family Health Strategy**					
	≥ 30.0%	-	-	1	-
	< 30.0%	-	-	1.12	1.08–1.14
**TB cure rate**					
	> 10.0%	-	-	1	-
	≤ 10.0%	-	-	2.87	2.65–3.12
**Rate of hospitalization TB**					
	> 10.0%	-	-	1	-
	≤ 10.0%	-	-	1.22	1.14–1.32
**Proportion of older men – 65 years and over**				
	< 4.0%	-	-	1	-
	≥ 4.0%	-	-	1.41	1.30–1.53

Source: Mortality Information System. SINAN / Datasus / Ministry of Health.

1 All forms.

2 Categorical model.

3 They refer to 1,614 municipalities that had better information quality.

RR = relative risk; TB = tuberculosis; HIV = human immunodeficiency virus; CI = confidence interval; LC = living conditions.

A statistically significant effect was observed for municipalities with a proportion of TB-HIV coinfection of > 5.0%, where RR = 1.32 (95%CI = 1.24–1.42). The mean risk of dying from TB in municipalities with a proportion of elderly patients > 4.0% was 41.0% (RR = 1.41; 95%CI = 1.30–1.53).

In municipalities where the FHS coverage was below 30.0%, the risk of death from this disease was 1.12 times higher than in those with a mean coverage greater than or equal to 30.0% (95%CI = 1.08–1.14). Municipalities where the cure rates for TB treatment were below 10.0% had an RR = 2.87 (95%CI = 2.65–3.12). In municipalities with a treatment dropout rate of > 5.0%, the RR = 1.33 (95%CI = 1.25–1.42). Areas with hospitalization rates for TB of < 10.0% presented an RR of 1.22 (95%CI = 1.14–1.32).

## DISCUSSION

The findings of this longitudinal study of spatial and temporal aggregates revealed that, from 2002 to 2015, the risk of dying from TB in the municipalities analyzed decreased by > 23% and that the improvement in LC in this period produced an overall reduction of 11% in the relative risk of death from TB. This protective effect remained even after adjusting for other important determinants and was higher in the strata of municipalities with low LC than in those with intermediate LC.

These results can be interpreted in light of social determinants and health inequalities.^12,26^ The differences observed in the risk of TB mortality between areas, populations, and social groups result from heterogeneity in the level of social development, income distribution, access to health resources, basic sanitation, education, and other LC determinants. From this perspective, TB can be spread unequally, both in the urban space and between subjects, due to its inclusion in the social reproduction process.^13^ Thus, in countries marked by poverty and marginalization, thousands of people are disproportionally and heavily affected by TB due to its strong social determination, particularly regarding the social inequality that predominates, as studies show, in countries with different living and income conditions, such as the Philippines,^27^ England,^28^ South Korea,^29^ and China.^30^ These characteristics can be observed in Brazil, given its economic development model is characterized by high inequality, social exclusion, and insufficient political and financial investments.^15,17^

Concomitantly, with the improvement in the population's LC in the municipalities studied, there was a reduction in treatment dropouts and hospitalizations for TB, which, in the continuous regression model, were not associated with this mortality in 2002–2015. Increased FHS coverage, in turn, had a protective effect.

These results provide evidence of the importance of public policies with multisector coverage, which contribute to improving the population's LC and health to promote health, prevent disease, and reduce mortality. On the other hand, there was an increase in the proportion of TB-HIV co-infection in the older adult population, which are important risk factors for this mortality and were shown to be associated with the outcome studied in the aforementioned regression model.

TB-HIV co-infection is a public health problem with high rates of occurrence worldwide, and it is related to the social determinants of health by systematically affecting more vulnerable populations, thus raising TB mortality indicators.^31,32^ Regarding the increased risk of TB death among the elderly, the displacement of the incidence of this disease to the elderly population stands out, highlighting the difficulty of diagnosing the illness in this age group, which may determine its high mortality,^33^ TB in the elderly is expressed as the resurgence of long inactive infection, as well as being due to the greater vulnerability of this population to reinfection.^34^ This greater vulnerability may be due to aging, relapses, difficult response to treatment, trivialization of symptoms, and immune system deficiency due to advanced age. These findings show the need for the elderly to receive greater attention from health services and professionals, not only for the early identification of TB but also for monitoring to reduce complications and deaths.^35^

Another fundamental point to discuss relates to the significant effect of the FHS, which presented a mean overall impact of 3% in reducing TB mortality, as observed by Souza.^36^ This strategy has a high level of decentralization and coverage, facilitating access to the health system and providing higher-quality care to TB patients. Many of these actions, such as early diagnosis of the disease, home treatment and visits, bacillus Calmette–Guéri vaccination, and anti-HIV testing, are performed using FHS units and may have consistently contributed to reducing mortality from this cause in the country.^37^

It is worth mentioning that since the 1980s, there has been a reduction in TB mortality in Brazil.^38^ However, its levels are still far from those of developed countries, which record mean values of 0.1/100 thousand inhabitants.^39^ Moreover, it is worth highlighting that the pace of this fall slowed after the advent of HIV in the country.

It is important to remember that Brazil has undergone profound political, economic, and social transformations geared toward less favored populations to reduce poverty in the country and promote better LC for these populations especially,^40^ which may explain the greater impact on the poorest populations. Studies indicate that the initiatives implemented and the progress achieved by the social programs in the country, as well as by the conditional income transfer programs for families living in poverty, such as the *Bolsa Familia* and Eradication of Child Labor programs, have generally promoted a significant improvement in LC.^41^ Income transfer programs play a fundamental role in reducing poverty and improving LC since they enable improvements in income, which can be used for housing, food, and nutritional security.^42,43^

Attention should be drawn to the fact that the use of secondary data may constitute a potential limitation of this study, given that they present restrictions in terms of quality, coverage, completeness, and validity. In addition, the study's 2000–2015 time frame was another limiting factor owing to access to data up to more recent periods. It is also worth noting that variables such as alcoholism, malnutrition, and mental illnesses, among other determinants of TB mortality, were not included in the regression model. However, we sought to minimize this restrictive effect by including in the study only municipalities with better quantity and quality of information. Moreover, the fact that all indicators employed presented a similar evolution to that observed in Brazil strengthens the results of this study.

## CONCLUSION

This study suggests that the improvement in the LC of the Brazilian population from 2002 to 2015 contributed to the reduction in TB mortality, especially in the stratum of low-LC municipalities, as the social interventions were primarily directed toward populations living in poverty and extreme poverty. Therefore, we must consider the social determinants of the disease and intersectoral strategies as priorities for a more significant reduction in TB mortality in Brazil. It is also evident that alongside the adoption of measures that provide access to adequate quantity and quality services for the population, the implementation of greater and continuous investments from other sectors seeking to reduce poverty and improve education is imperative, as their effects will be positively reflected in the quality of life and health of human collectivities.
